# “The worst thing that has happened to me”: Healthcare and social services professionals confronting death during the COVID-19 crisis

**DOI:** 10.3389/fpubh.2022.957173

**Published:** 2022-07-26

**Authors:** Carlos Hernández-Fernández, Carmen Meneses-Falcón

**Affiliations:** Department of Sociology and Social Work, Universidad Pontificia Comillas, Madrid, Spain

**Keywords:** death, COVID-19, social services and healthcare professionals, burnout, death anxiety

## Abstract

**Objectives:**

This study analyzes the subjective emotional impact COVID-19 deaths have had on healthcare, social services, and funeral services professionals, it explores the different implications, and analyzes the different reactions of health and social care professionals and funeral professionals to the volume of deaths.

**Methods:**

This work is based on a qualitative, phenomenological, and interpretative approach through in-depth interviews with 42 informants, including 36 social and healthcare professionals, as well as 6 family members of those who died from COVID-19 in Madrid. The interviews were processed through a qualitative, interpretative, categorical analysis.

**Results:**

Healthcare professionals were overexposed to a significant number of deaths under dramatic circumstances. Many of these professionals had difficulties processing their experiences and expressed the need for psychological help. The fact that certain professionals had previous exposure to high mortality rates was not a protective factor. Some coping differences were seen between healthcare professionals and professionals dedicated to the care of the deceased (undertakers or firemen), particularly in the degree to which they personalized the care they provided.

**Conclusion:**

The overexposure to death with the circumstances that existed during the state of emergency had a significant emotional impact on the professionals, which can lead to mental health problems in the near term.

## Introduction

Worldwide over 2,500,000 people died from COVID-19 between the end of 2019 and March 2020 (1). The number of infected and deaths was especially high in Italy and Spain.

In Spain, 152,230 deaths from illnesses occurred between the months of March and May 2020, of which 45,684 were caused by the COVID-19 virus (2), a 44.8% increase in deaths compared to 2019.

Hospitals were overflowing and had to convert operating rooms into ICUs and common spaces, like gyms and waiting rooms, into treatment areas.

Protocols were activated for hospitals and senior residences, prohibiting visitors and preventing family members from accompanying their loved ones. Healthcare and social service workers were the only companions and witnesses to the deaths of their patients.

The systems for managing the deceased broke down and funeral homes were overflowing (3). Improvised morgues were created, and military and fire service personnel were mobilized to transport bodies.

Front-line professionals had to respond in extraordinary circumstances never previously experienced and lived intimately with an inordinate number of deaths. These professionals were the last ones to see patients alive (4) which means they had to provide emotional support (5) and accompany patients through death to the extent possible.

Some healthcare and social services workers who were not used to witnessing deaths, either because of their specialty or usual patient type, were newly exposed to death and dying in a dramatic way.

The death anxiety described by Tomer et al. (6) is frequent, for example, in palliative care professionals accustomed to witnessing the death of patients, now appears in many other health care professionals, generating burnout, stress and emotional fatigue (7). It is also possible to find medium and long term effects such as those described in reviews on the intervention of professionals in humanitarian catastrophes (8, 9).

COVID-19 is an infectious disease, with which the professionals must co-exist. These professionals, were exposed to death at a much higher rate, which lead to a fear of death both that of their own and of their family members (10). On top of this, they suffered immense stress caused by fear of deterioration and death of their patients (11).

This study aims to (a) analyze the subjective emotional impact that COVID-19 deaths have had on healthcare, social service and funeral service professionals, (b) explore the different implications for one type of professional versus another, and (c) to analyze the different reactions of health and social care professionals and funeral professionals to the volume of deaths.

This research is particularly relevant as it was carried out just after the most critical months of the pandemic in one of the hardest hit cities. The study highlights the impact of the deaths that occurred during the COVID pandemic on professionals and their possible consequences. It also aims to highlight the need for prevention plans for future events.

## Methods

### Design

With the aim of uncovering inherently subjective aspects such as the meanings, perceptions, and emotions experienced in critical situations like the pandemic, a qualitative methodology was used for this work (12). Specifically, phenomenological and interpretative interview techniques were used. In some of the interviews, where the aim was to inquire about lived experience, a phenomenological approach was applied (13). In other cases, in which the intention was to collect a diversity of experiences, meanings and emotions, a more thematic and interpretative approach was applied. The approach of the interview depended, therefore, on the type of informant and his or her role in relation to the topic discussed in the study. Table 1 specifies the type of information on the front page for each respondent and whether or not this information is related to their direct experience.

**Table 1:** Data sheet of the subjects interviewed and categories.

**Interview with professionals**	**Role**	**Reflected categories**	**Principal contributions**
**Hospital employers**			
IP01	Medical Director	A,C,E,F,G,H,K, L,M,N,O,Q	Overview of the experience of professionals in the hospital. Testimony of their own experience. Information on protocols and decisions.
IP02	Psychologist	A,C,E,G,H,M,N, O,P	Testimony of their own experience in accompanying the dying and in the relationship with family members.
IP03	Patient Experience Department Representative	A,C,H,I,J,L,M,N, O,Q	Testimony of their own experience in accompanying patients and informing family members about the death of their loved ones.
IP04	Nurse	A,B,C,G,H,J,K,L,M,N,Ñ,O,P,Q	Testimony of their personal experience in relation to the death of patients
IP05	Nurse	A,B,C,D,E,G,H, I,J,K,L,N,Ñ,P,Q, R	Testimony of their personal experience in relation to the death of patients
IP06	Doctor	A,B,C,G,H,K,L, M,N,Ñ,O,R,	Testimony of their personal experience in relation to the death of patients
IP07	Doctor	A,B,C,D,E,F,G, H,K,J,L,M,O,Ñ, O,P,Q,R	Testimony of their own experience with facing the death of patients and reactions of family members
IP08	Social worker	A,E,F,L,	His role was not directly related to deaths, but to coordinating patient care and facilitating contact with families.
IP09	Chaplain	A,H,I,J,L	Testimony of their own experience with facing the death of patients and reactions of family members
Senior residence employees			
IP10	Director and Owner	A,G,H,I,J,K,L, M,N,O	Overview of the experience of professionals in residences. Testimony of their own experience. Information on protocols and decision-making in nursing homes.
IP11	Orderly	A,E,G,H,I,J,L, M,N,O,Q	Testimony of their personal experience in relation to the death of patients
IP12	Social Worker	A,E,G,H,I,J,L, M,O,	Testimony of their own experience with facing the death of patients and reactions of family members
IP13	Psychologist	A,C,E,G,H,I,J,L, M,O,P,Q	Testimony of their personal experience in relation to the death of patients
IP14	Communication Director	A,C,E,G,H,I,J ,K,L,M,N,O,R,P,Q	General overview of the experiences of professionals working in residences. Testimony of their own experience. Information on protocols in residences.
IP15	Social Worker and Sales Manager	A,C,E,G,H,I,K,J,L, M,N,O,R,P,Q	Testimony of their own experience as well as the experiences of colleagues.
IP16	Orderly Coordinator	A,C,E,H,I,L,J, M,N,P,Q	Testimony of their personal experience in relation to the death of patients
IP17	Orderly	A,C,E,H,I,J,L, M,N,P,Q	Testimony of their personal experience in relation to the death of patients
IP18	Director of Residence	A,B,C,H,G,N,Ñ, P,Q,R	General overview of the experiences of professionals working in residences. Testimony of their personal experience.
IP19	Orderly	A,C,E,F,H,I,L, M,N,Ñ,P,R	Testimony of their personal experience in relation to the death of patients
IP20	Chaplain	A,C,E,G,H,N,O	Testimony of their personal experience in relation to the death of patients
Funeral services professionals			
IP21	General Secretary and Secretary of the Board of Directors	A,C,H,L,N,Ñ,O, Q,R	Contextualization of work in a funeral home. Overview of the experience of professionals who work in funeral homes. Testimony of their own experience.
IP22	Sales Director	B,C,H,I,K,L,N,Ñ, O,Q,R	Testimonio de la propia experiencia en relación con la muerte, la información a familiares y al tratamiento de cadáveres.
IP23	Hearse Driver and Mortician	C,H,K,L,N,O,Q	Testimony of their own experience in relation to death and the treatment of corpses.
IP24	Customer Service Representative	B,C,H,I,L,N,Ñ,Q	Testimonio de la propia experiencia en relación con la muerte y la información a familiares
IP25	Public Relations Representative	B,C,H,I,L,N,Ñ,Q	Testimonio de la propia experiencia en relación con la muerte y la información a familiares
IP26	Chaplain	A,H,I,K,O,Q	Testimony of one's own experience in relation to death and rituals.
IP27	Public Relations Representative and Crematorium Technician	B,C,F,H,I,K,L,N, Ñ,O,P,Q,R	Testimonio de la propia experiencia ante la muerte y relación e información a familiares en crematorios.
IP28	Undertaker	A,B,C,H,I,K,L, N,Ñ,O,Q	Testimonio de la propia experiencia ante la muerte y relación e información a familiares en entierros.
IP29	Public Relations Representative	A,B,C,H,I,K,L, N,Ñ,O,Q	Testimonio de la propia experiencia ante la muerte y relación e información a familiares.
Emergency services professionals			
IP30	Doctor	A,C,D,G,H,K,J, L,M,N,Ñ,P	Testimony of their own experience in relation to deaths occurring at home.
IP31	Nurse	A,C,D,G,H,J,K, L,M,N,Ñ,P	Testimony of their own experience in relation to deaths occurring at home.
Emergency social workers			
IP32	Volunteer Social Worker		Testimony of own experience in communicating with families.
IP33	Volunteer Social Worker	A,H,H,Ñ,Q,P	Testimony of own experience in communicating with families.
Others (collection of corpses)			
IP34	Firefighter	B,C,G,H,I,L,M, N,Ñ,O,Q,R	Testimony of their own experience in relation to death and the treatment of corpses.
IP35	Firefighter	B,C,G,H,L,M,N, Ñ,O,Q,R	Testimony of their own experience in relation to death and the treatment of corpses.
IP36	Priest Improvised Morgue	A,E,F,H,N,P,	Testimony of their own experience in relation to death in a completely new environment.
Interview with familiars			
IF01	Daughter of Deceased	B,H,J,N,O	Narrative on the perception of the work and mood of healthcare professionals working in hospitals and communications with them.
IF02	Daughter of Deceased	C,F,H,J,L,N,	Narrative on the perception of the work and mood of healthcare professionals working in hospitals and communications with them.
IF03	Daughter of Deceased	B,C,G,H,J,L,M,N,	Narrative on the perception of the work and mood of healthcare professionals working in hospitals and communications with them.
IF04	Daughter of Deceased	A,E,F,G,H,J,K,L,N,Ñ,	Narrative on the perception of the work and mood of healthcare professionals working in hospitals and communications with them. Narrative about the relationship with funeral professionals.
IF05	Granddaughter of Deceased	A,E,F,G,H,J,L,N	Narrative on the perception of the work and mood of healthcare professionals working in hospitals and communications with them.
IF06	Wife of Deceased	A,B,F,H,J,M,N,	Narrative on the perception of the work and mood of healthcare professionals working in hospitals and communications with them. Narrative on the perception of the work and mood of funeral services professionals.

The experience of the participants who had to face, in one way or another, situations of death during the state of emergency is explored in depth, collecting their feelings, perceptions, and thoughts, and observing how they gave meaning to what they experienced. The interview offers a contextualized view of the experience, allowing one to historically and socially frame personal experiences and thus understand the social processes that may underlie subjective evaluations or interpretations (14).

### Recruitment and sampling

The study was carried out in Madrid, a city with one of the highest demands for emergency services and healthcare provision between March 2020 and May 2020 (15). To ensure a diversity of perspectives was acquired, 42 informants of different types were interviewed incorporating: (a) nine hospital employees including doctors, nurses, social workers, psychologists and chaplains; (b) eleven senior residence employees including management, psychologists, social workers, chaplains and orderlies; (c) two emergency services professionals, one a doctor and the other a nurse; (d) nine funeral services professionals across all functions including management, office administration, sales, customer service, transport, chaplains, crematorium technicians, and undertakers; (e) two firefighters; (f) six relatives of the deceased; (g) two emergency social worker and (h) one priest of improvised morgue (Table 1). In the case of the professionals, informants were selected who had different roles and worked in distinct types of institutions. The aim of this selection was to have three types of informants: i. Professionals who worked with the deceased, ii. Professionals who worked with the deceased and/or their relatives and iii. Relatives of the deceased who had contact with the professionals. All participants were contacted by telephone, the project was explained, and their collaboration was requested. The interviews were carried out progressively, following the theoretical sampling model of Glaser and Strauss (16), utilizing the constant comparison between each type of informant, and seeking distinctive aspects in newly selected informants, or to augment central analysis categories that required greater depth; finally, the research questions and objectives guided the inquiry process and the search for new observations and interviewees. When information received was repeated over and over, the information required to fulfill the objectives was considered to have reached a saturation point. Interviews were conducted between July and November 2020. One of them was conducted in writing and seven by videoconference due to pandemic restrictions. The rest were conducted in person, and all were recorded. The interviews were approached as a conversation, following Kvale (17), around three dimensions: (a) the impact of being overexposed to death, (b) their experience with the deaths compared to previous stages of their lives, and (c) how they assimilated and processed the experience.

All of the interviews were conducted in Spanish and this article was written in Spanish, then later translated.

### Ethical considerations

Considering the sensitivity of topics involved in this research, compliance with the appropriate ethical requirements was maintained, under the supervision of the university Ethics Committee, who issued a report of approval. All participants were informed of the objectives of the research study, the sources of financing and the planned use of the results. Informed consent was solicited, and informants were notified that their participation was voluntary. Permission for audio recording was also requested. Confidentiality was guaranteed through a confidentiality agreement.

### Data analysis

Data Analysis. After the verbatim transcription of all the interviews, the analysis began with the support of the Nvivo 12 plus program, which facilitated categorization and codification. The analysis was carried out in three phases: exploration and discovery phase, categorization and codification, and interpretation (18). The participants' discourses were examined *via* a categorical analysis that considered both content and discourse analysis (19). First, the language used was explored, taking into account the words and phrases used and the sentiment associated with them (20). Secondly, the analysis focused in on the meanings associated with death and the farewell to close relatives. The development of the analytical categories and the codification of the interviews were central to this stage (Tables 1, 2) (21). The last step of analysis was the interpretation and association of meanings with the circumstances and contexts in which they took place. The main strategies of rigor and quality criteria associated with qualitative research were applied (22). Reflexibility was used in the data collection process, as well as content saturation and key categories; to prevent biases in the first author's interpretations, the second author reviewed the results and analysis for dependability and confirmability (23).

**Table 2:** Categories.

**Indicator**	**Category**	**Description**
A	Accompanying	Narratives on how to accompany dying patients and families.
B	Mutual support	Experiences on supportive relationships between professionals.
C	Assimilation	Statements expressed about the way of assimilating what happened and the evolution of this process at the time of the interviews.
D	Self-protection	Explicit manifestations of self-protection strategies by professionals.
E	Death awareness	Expressions about how their lived experience connects with their awareness of their own death.
F	Contact	Narratives and accounts of experiences of physical contact between professionals and patients in the moments before death.
G	Life and death decisions	Stories and reflections on decision-making about the administration of therapeutic resources and medical triage.
H	Emotions	Expressions of emotions and feelings experienced before death and illness, both in family members and professionals.
I	Professional effort	Narratives about professional overexertion. Refers to hours worked, physical or mental effort, hours of sleep, etc...
J	Moment of death	Narratives explaining the moment of death and the circumstances surrounding it.
K	War or disaster situations	Comparisons made in the interviews with situations of war or humanitarian disasters.
L	Informing family members	The professionals tell how they informed relatives about details related to the death of their loved one or about the treatment of the corpse. Family members talk about receiving the news.
M	Unexpected death	Verbalizations about sudden and unexpected deaths and the reactions of the bereaved.
N	Organization of corpses	Comments on the organization of cadavers in an environment of chaos and its impact on the work of professionals.
Ñ	Care demands	Comments on workload and care demands
O	Healthcare and mortuary protocols	Refers to the protocols established by the authorities that prevented or allowed the farewells, or to sit vigil with the corpse, as well as the occasions in which they were not complied with.
p	Relationship to death	Instances that highlight the relationship of the interviewees with death.
Q	Number of deceased	Verbalizations about the disproportionate volume of deaths and their impact on professionals.
R	Job title and function	Clarifications on the organization and functions of professionals.

The results are presented by addressing six themes: The psychological cost on professionals, the different reactions and coping strategies, the professionals' personal relationship with death, the emotional impact of decision-making, the difficulty in processing the experience and repercussions on mental health, and the family members' perceptions about professionals.

## Findings

Between March 2020 and May of 2020, during the state of emergency decreed by Spain, healthcare professionals in Madrid confronted demands for their services and an accumulation of deaths at levels never previously experienced.

Health care professionals were subjected to levels of emotional and/or physical stress and exhaustion that are proving difficult to process months later and some of them expressed a need for psychological help. Humanizing and personalizing care during the pandemic, as well as the need to make decisions about resource management and life support were significant sources of emotional stress for healthcare and social services workers. Professionals working to manage and process cadavers report greater physical rather than emotional exhaustion. The challenging work of the professionals in these circumstances was recognized and highly valued by relatives of the deceased. All verbatin, which support the results, are shown in Table 3.

**Table 3:** Verbatin of the interviews illustrating the results obtained, classified into responses from professionals and responses from family members: Madrid. Spain 2021.

	**Verbatim transcriptions**
	Professionals
VP1	*It was a brutal emotional load. And physically I don't know, there were many times I didn't know where we found the strength to continue on*. (IP12)
VP2	*There is always a case that hits you a little harder. Well, at least that is what I say. Then you shed two tears, you immediately start thinking about something else and it's over*. (IP27)
VP3	*Yes, it psychologically scars, scars, (...), and it especially scars psychologically because it is a family's pain that they are passing on to you and you empathize with them, even if from a distance*. (IP24)
VP4	*Look, the truth is that I used to leave shifts crying every day, in the car, I would get in the car, until I got to the car I was cheering people up, but when I got in the car I would start crying like a baby until I got home*. (IP30)
VP5	*For me this has been the worst thing I have ever experienced in my job. It has been terrible. When we had the first positive case and they started to raise the alarm, that was terrifying*. (IP12)
VP6	*We are not accustomed to this in Madrid, or at least I am not, to people dying without you being able to offer them everything you have. I have gone to other places in summer, I have been working in other places as a doctor, places where diabetics die because they don't have a fridge, and when you are there, you accept it for what it is, but you never think that it could happen in Spain*. (IP06)
VP7	*Look, what I have processed a lot is the agony of dying alone. The agony of not being able to say goodbye in the final moments you are alive..*. (IP14)
VP8	*So, it was a feeling of seeing patients dying and not being able to help them, and well, it has been and was horrible, horrible, horrible*. (IP07)
VP9	*One especially odd thing that happened to me was when we were very calm, I went into a room one night and stumbled upon a cadaver on the ground, and this room was already occupied by another person and so, I entered into this room and I got angry, like a rage came over me for a few seconds, I mean, what do I do with anger in this context? It was, like, a very rare thing and very disjointed, and this is how the anxiety came and this has happened to many healthcare workers*. (IP04)
VP10	*I know that it sounds very hard and very cold, and perhaps what I am going to say is appalling. But you were protecting yourself, saying: “This is not a person. It does not concern me, I do not care, I attend to them, I save their life if I can, I treat them, I do everything I can, but if they die, I don't want to know.”* (IP05)
VP11	*That wasn't the worst for me, the worst that I experienced personally was when a patient died you activated the protocol (...) then we had to write the name with permanent (marker) on the sheet... that was the worst part for me because I did not learn the name of my patients. I am very... in normal conditions I didn't take bed 1 and 2, I took patient so-and-so by name, but no, in this case I couldn't. Today it was Francisco María, and tomorrow it was Pascual. Well...for what? (silence)* (IP05)
VP12	*And then one of the other things that happened is that everyone, doctors included, well, of course, you have a much closer bond with the patient who tells you their stories, and, so you knew much more about the patients than you might in the usual pace of life in the hospital when, perhaps, they are there for less time, or a family member is there with them. The people speak to you about their grandchildren, they tell you about such and such, (...) So every death that occurs...well, of course, they did not discharge number 103, Leandro has died, Leandro whose wife was admitted first, whose wife got out but not him. So, he has a story. Maybe the right thing would be not that you get used to it, but that it leaves less of an impression than at the beginning because there have been so many, but no*. (IP01)
VP13	*And saying goodbye to them and telling them that you are here, that you have been here, because at the end, they, I am not going to say that they love you more than they love their family, (...) but if we are with them all day long, taking care of them all day long, showering them, bathing them, helping them eat, helping them walk...well, ultimately they end up loving you as if you are a member of their family*. (IP11)
VP14	*We are used to putting on our armor and being up and running every day, and this has really made us take off the armor...to say, “Shit. This isn't normal.”* (IP22)
VP15	*We are used to responding to what happens to someone else, but in this case, it is happening to someone else, but at the same time it could happen to you, it therefore causes a mix of emotions*. (IP21)
VP16	*You know, I was more worried about what was going on at home, than the impact picking up the bodies would have on me. Because as far as it is a job, you do it and that's it. And for that reason, it has had practically no impact on me. What impacted me was concern for my father, that the next day I could very well see him under one of the shrouds*. (IP25)
VP17	*And then, well, Dantean scenes. (...) A colleague said, “They have them (the bodies) on top of the tables.” It is like, where else are they going to put them? There is no other way to do it. Often, they were on the floor..*. (IP34)
VP18	*It was like, 'Shit, this seems like 'The Walking Dead' or I don't know,' it was like a very weird movie, you would arrive to pick them up...but of course, we went, it was like we were delivery drivers for, I don't know, Amazon, you know?... we went to a place, we did the pick-up and later we took them to the centers where we were supposed to take them*. (IP34)
VP19	*Well, any one of the people there could have been my father (...) It was dreadful, because at the time I was living with my father, and my father turned 62 yesterday. It made me terrified. I would arrive, grab the plate, and go into my bedroom*. (IP05)
VP20	*It has made us think and begin to prepare our affairs in case you die. So, I'm telling you things like that, or regarding leaving things arranged in your life, this, and*
	**Professionals**
	*that, and so on, ehhh... The fear of infecting your family, the fear of infecting your father, the fear of infecting everyone around you*. (IP30)
VP21	*As soon as they let me travel the first thing I did was go see my parents. And it is very clear where the things are for when my parents die, the green folder as my Mother says, the dead people's telephone, as my Mother says. (Laughing)* (IP04)
VP22	*Well, yes, it has changed, my concept of death, of living life, it is like that, people think that death is the end of life, and it is not, but it is part of life*. (IP04)
VP23	*In other words, we live totally oblivious to it, as if it will never happen. This has been a reality check in that sense because there has indeed been a 180% excess mortality rate in the community of Madrid. It is astounding. It has made us much more aware of the fact that we do indeed die*. (IP02)
VP24	*What I have learned about the topic of death is that there is a taboo about death, it is not talked about, especially in our industry, it is always hidden. So, from the (ethics) committee, yes, we have spoken about it, and I believe that we should improve the implementation in our service offerings as well, this issue of advanced directives. So, I think this should help us to begin to deal with all these issues more naturally*. (IP15)
VP25	*One must change the chip, because if not, we couldn't work here. How can you begin to empathize with all of the families, or allow yourself to start thinking that you work with death, with the pain, with the crying, at the end I believe you couldn't handle it, you would end up depressed*. (IP27)
VP26	*I have a very close relationship with my parents, and just thinking that something could happen to them, that at some point something is going to happen to them because they are already 80 years old, well, I don't handle it very well...it is that losing.... seeing that death is horrible, but, well, I don't know, I try not to think about death*. (IP07)
VP27	*A patient dies and you are left depleted, then comes a moment of pain, but you ignore it. (...) You try not to learn their name, hope that the family does not call you, and..*. (IP05)
VP28	*The medical part, in terms of attending to the people, was terrible, terrible because knowing that the hospitals were overflowing, sometimes we had to make decisions which are hmmm, it is not politically correct to say it, but we have had to let people die at home who in other circumstances could have, they might have been able to continue on*. (IP31)
VP29	*Because it was people that were still very alive, because the feeling is that you can't do everything that you should have been able to do. Because the people that are admitted now have the right to a ventilator (...), but the people in March and April didn't have that*. (IP06)
VP30	*In other words, many people were dying that ethically, should not have died..*. (IP07)
VP31	*It is terrible because these people need healthcare attention just as you and I might need it. What happens? Because they are elderly you deny it to them? No, there is no right to do that, because they are equal people (cries). So, why were these people denied that?* (IP12)
VP32	*And that we are the murderers. I, in a chat of school mothers, the theme came up and I had to speak up. I stayed silent. “It is a shame that there are murderers who didn't take them (dying patients in senior residences) to the hospitals.”* (IP15)
VP33	*No, this is never going to be processed. It will stay here forever, no matter how much it is discussed, whether you speak to a professional, it is not going to matter, what we have experienced, is experienced, I believe. No...it is something that will stay with us..*. (IP17)
VP34	*And it is that you go out for a run and all of a sudden you feel like crying, and it lasts 5 minutes. And you return home like, what just happened? Or suddenly you can't sleep again at night*. (IP04)
VP35	*This has an impact. I already had a time when I went to a psychologist, a psychologist who was a friend of the family and she had told me “You seem to be a strong person...but it affects you...”* (IP19)
VP36	*We spoke about what we had experienced, how it seemed like so much more time had passed, (...), that we didn't really know how to act with the family or what to say to them, that we were not prepared for that, what we were experiencing with our own families and such*. (IP32)
VP37	*I believe that they (members of the department) have managed through it very well, uh, at a personal level. The Red Cross also came, two psychologists came here, to do a little therapy with us for the emotional effects that we might have, perhaps, negative (effects) from this and the truth is that, not that there has been resistance, but rather that we have listened and so on, and the feeling for me, because it coincided with three or four of my shifts is that...it is not that they haven't done their job it is that it wasn't very necessary. There was no need. (...) They (members of the department) have managed well, those who had problems with taking it home with them were more afraid but well, quite well, yes..*. (IP34)
**Family members**	
VF01	*And after a little while SAMUR (emergency services) came and I tried to tell them to do this, with the defibrillator and such, and the man looked at me and said, “My queen”—I will not forget these words—“My queen, he has left you.” This man was a real sweetheart, really. He was huge, big, with glasses, (...) and he told me, “He has left you; he has left you.“* (IF06)
VF02	*I started to really cry. The doctor began to cry, his tears were falling*. (IF04)
VF03	*What I understand about the toll the work takes, (...) and that they told me “Okay, okay, we understand you, but we can't keep up.” I got angry and I told them “I don't care, it is your job,” I told them, and later I regretted it a ton. They did what they could, but it made me so angry...but hey, it is what it is..*. (IF05)

1. **The psychological cost on professionals**. **“*I have worked in this profession for*** ***25 years and the truth is I have never experienced anything similar.”***

During the interviews, the professional caregivers (healthcare professionals, psychologists, social workers, etc.) in recounting their experiences during this stage of the pandemic and in relation to deaths, expressed a notable degree of distress and showed significant emotional exhaustion both verbally and non-verbally (VP1).

The professionals whose work involved direct contact with bodies of the deceased (funeral services professionals and fire-fighters), but who did not have direct contact with living patients or their relatives, referred more often to the volume of work and the extraordinary nature of the situation, but showed a greater emotional distance from the deceased and less psychological exhaustion (VP2).

The funeral services personnel that directly served family members of the deceased, did express a greater psychological toll, at levels similar to caregiving professionals, as compared to other funeral services colleagues (drivers, technicians, and undertakers), who had no direct family interaction (VP3).

The workers that had had contact with patients or their families, could not suppress their emotions during the interviews, and said they had cried daily during the months of confinement (VP4). They often stated that this is the worst experience of their professional lives (VP5) and some compared the situation to what transpires in disasters, wars and third world countries (VP6).

2. **Humanizing care and coping strategies**. **“*Patients must be touched when they*** ***are dying, you must be with them.”***

Despite the high number of deaths that occur in a typical nursing home or hospital, many healthcare and social services professionals indicated that they have never become accustomed to the phenomenon of death and were particularly impacted by the unique circumstances created by the pandemic, where patients died alone and without adequate care due to a lack of resources (VP7 and VP8). They recounted the dramatic way in which some of the deaths occurred, and the anxiety they still feel when reliving them (VP9).

The act of personalizing each patient and providing more humanizing care influenced professionals' emotional experience, causing greater psychological harm. Some professionals guarded against connecting personally with patients to protect themselves (VP10), as actions such as learning a patient's name or having to write it on the shroud after death could deliver an emotional shock (VP11).

While some professionals protected themselves by not personalizing patients and despite the tremendous workloads they had to manage, in some cases the professional-patient relationship became more intense than in periods prior to the pandemic, especially with the absence of family members. In these cases, the deaths could be even more painful for the professionals (VP12). This effect was magnified for senior home personnel given their close relationships with the residents, who could come to consider the professionals part of their family (VP13).

The professionals that historically worked with the deceased, but not with living patients or families, generally seemed more accustomed to the phenomenon of death, although they also recognized that the circumstances of the pandemic were anomalous, and they had more intense experiences in their work than before the pandemic (VP14).

For these professionals the difference from the pre-pandemic era resided not only in the volume of work, but also in the fact that they were managing a situation in which they themselves could become victims (VP15). Above all, the impact of the situation was especially notable in their concern for what might happen to their families (VP16), rather than the significant number of corpses seen in residences and hospitals, described as “Dantean” scenes (VP17). In verbalizing their thoughts, they used comparisons that allowed them to narrate the situation with an outsider perspective, even with a touch of levity (VP18).

3. **Professionals' personal relationship with death**. **“*But nothing is more*** ***certain, nor more denied than death.”***

During the most intense months, healthcare and social services professionals were afraid of the virus and of death, and they were especially afraid for their families (VP19).

In many cases this fear, and the overexposure to dying forced them to reconsider their relationship with death and think about it in different ways. Some thought about the need to prepare for their own death (VP20), to address related issues with their family members (VP21), or to enjoy life more (VP22).

Several professionals commented that, after this experience, society as a whole and professionals particularly have become more conscious about the reality of death and have begun to see it as something that exists closer to home (VP23). They also highlighted how there is an increasing need to have more proactive conversations around related issues in senior residences and hospitals (VP24).

However, other workers, both in healthcare and funeral homes, signaled that they prefer not to think about death. For them this is a personal coping mechanism (VP25), to avoid having to confront the possibility of family members' deaths in the future (VP26), or as a method of self-protection to avoid suffering when their patients die (VP27).

4. **Decision-making and emotional exhaustion**. **“*So, you attended to one and the*** ***other could die.”***

One of the major sources of stress, helplessness and frustration was the need to constantly choose who to prioritize for treatment (VP28) or which patients should be admitted to the ICU and which should not. These decisions made healthcare workers feel that they were letting people die who could have survived (VP29), which provoked serious concerns around ethics and crises of conscience (VP30). This frustration was especially intense in senior residences, where the professionals saw how residents were denied treatment (VP31), frustration that was compounded by a sense of being accused by the public of being personally responsible for residents' deaths (VP32).

5. **Difficulty in processing the experience and repercussions on mental health** **“*No, this will never be fully processed. It will stay with you forever.”***

When healthcare and social services professionals were asked about their current state of mind, many commented on their difficulty with processing what they had experienced (VP33), they described symptoms associated with possible post-traumatic stress (VP34), and they highlighted the need for psychological help to overcome the trauma that the situation is causing them (VP35). They emphasized that during the most difficult months of the pandemic, mutual support among colleagues had been fundamental to coping with the situation. (VP36). In the case of firefighters and funeral services professionals, conversations among peers were described as useful but they did not express a need for psychological support (VP37).

6. **Family members' perceptions about professionals**. **“*The doctor began to cry*,** ***his tears were falling.”***

It is worth mentioning that relatives of the deceased commented that even in the most dramatic moments, the social and health care professionals acted with sensitivity and humanity despite the demands for their services and the exceptional nature of the situation. (VF01). These family members were witnesses to the tears and helplessness of many healthcare providers and to their emotional state (VF02), and it is something that was valued positively. They acknowledged that at certain times it was difficult to understand the professionals' reactions, or the lack of information being provided, but afterwards family members exhibited empathy and gratitude for the professionals (VF03).

## Discussion

The number of deaths that healthcare and social services professionals, as well as funeral services professionals, have had to deal with during the state of emergency has been inordinate and unexpected (3). It might be presumed that healthcare and social services professionals, who routinely witness deaths in their work, may be accustomed to deaths and are therefore more sensitized (24), and that these professionals' continuous contact with death allows them to create strategies to facilitate future contact with death (25). However, the results of this study indicate that, when faced with the COVID-19 crisis, professionals were not able to get used to the unique circumstances, and the emotional impact caused by the deaths was elevated, including in those professionals who work in high-mortality environments like ICUs, emergency rooms, palliative care, and senior residences.

As this study has shown and according to Chocarro (26), depersonalizing the patient, avoiding conversations, or avoiding learning patients' names are coping strategies used by some professionals. However, it has been found that this has not always been possible or effective, since, as indicated by Ferrán and Barrientos-Trigo (5), during the pandemic, professionals had to supplement, to the extent possible, the emotional support required by a dying patient that would otherwise fall to family members, or that might be alleviated by the support of other patients, especially in senior residences. In a situation of scarce resources, with very difficult working conditions similar to those in developing countries, or generated by disasters or wars, healthcare professionals have had to use their imagination to accompany and care for the sick as described by Torre (27). This assumption of emotional care for patients has contributed to later separation anxiety among personnel (28) and difficulty in coping with death. On the other hand, the pressure to provide care was so high and the deaths that resulted occurred in such a dramatic way that any coping strategy could prove to be insufficient. It follows that many professionals are now in psychological treatment or say that they need it (29). Studies show that health professionals, who have worked during the first months of the epidemic, have experienced psychological symptoms such as stress, anxiety and depression, compassion fatigue and post-traumatic stress (30).

Without claiming to be a clinical assessment, it is found that, following the reviews carried out by Sakuma et al. (9) and Brooks et al. (8) the psychological and emotional effects that are noted in this study can be similar to those described in disaster situations and with humanitarian relief, such as emotional distress or compassion fatigue, among others.

One should not forget that the healthcare and social services professionals have been socialized through the same processes as the population they serve, and therefore dismiss the idea of death in the same way that the rest of the population does (26). Studies conducted before the pandemic (31) indicate that some healthcare workers demonstrate negative attitudes toward the concept of death and that this is one of the situations that regularly generates the most stress, among nursing staff for example (26). This research exposes the existence of these negative attitudes, which have been exacerbated by the impact of the deaths during the crisis. The professionals have narrated their difficulty in coming to terms with these deaths, even more so in an environment in which they considered that the deaths could have been avoided. For many healthcare professionals, death is not only something that isn't accepted, but also something that they prefer to avoid in their everyday thinking (32) in and during this crisis they have had to confront it daily. During the hardest months of the pandemic, death anxiety increases markedly among professionals (33).

The psychological distress for these workers is also caused by their perception of the risk of infection to themselves and their families, as previously indicated by Simione and Gnagnerella (34). So not only do they suffer with the deaths of their patients, but many of them, as this study indicates, connect the deaths of their patients with a fear of losing their loved ones.

Most of the research about psychological distress for healthcare and social services workers during the COVID-19 crisis does not refer explicitly to the relationship the healthcare professionals have with death, as evidenced by Bohlken et al. (35) and Spoorthy et al. (36) in their review of the literature. However, the present study considers that the professionals' prior experience with death is a determining factor for understanding their fears, emotions, and their need for psychological support.

With respect to social workers, we find few studies that speak of their relationship with death. Some, like that of Quinn-Lee et al. (37) carried out with palliative care social workers, affirm that exposure to death at work decreases the anxiety it generates. As has been suggested with respect to health care workers, this study indicates that such standardization is not applicable in times of pandemic crisis. On the other hand, Martínez-López et al. (38) point out that social workers in Spain have suffered high levels of anxiety about death during the pandemic, especially in relation to fearing the death of others and the process of death.

In the case of funeral services workers Van Overmeire and Bilsen (39) indicate that the COVID-19 crisis also generates a risk to their mental health, due to the number of funerals, the high demands of their job, and overexposure to death in the course of their work. This risk was not evident in the present study. Although the data indicate that the demands and workload were frequently mentioned factors for funeral services professionals, there was not similar evidence in the data for an overexposure to death. The same author indicated in a later study that compassion fatigue and burnout among funeral home personnel is lower than among healthcare professionals (40). Rodríguez-Rey et al. (41) also state that the psychological impact on protective services professionals has been lower than on health professionals. Previous studies also indicated that there is no relationship between exposure to death and mental health in these groups (42).

## Limitations

This study faced notable limitations due to the circumstances of the pandemic: (a) access to a wide range of healthcare professionals was difficult due to their ongoing workload as well as their state of mind; (b) the interviews were designed to be conducted in person, however some had to be conducted virtually making nonverbal communication and observations of nonverbal expressions difficult; (c) some professionals, especially social workers, were very reluctant to participate for fear of revealing particular professional situations experienced in their workplaces and (d) the great diversity of roles of the selected informants results in a heterogeneous sample.

## Implications

This study reveals the need to establish mental health surveillance measures for all frontline professionals who have worked with patients who have died during the most difficult months of the pandemic.

Supportive resources such as support groups and spaces for emotional healing should be strengthened.

It is necessary to expand the curricula of healthcare and social services professional training to include subjects that support development of coping skills for dealing with death, both in periods of crisis and in normal care provision, as well as expanding the bioethical view of death. It is furthermore advisable to promote initiatives whereby professionals and patients can talk about death to further normalize it.

This study opens the way for other research in the mental health field to consider the experience of death as an indicator of mental health and to study the real impact of this crisis, in the medium and long term, on healthcare and social services professionals as well as other professionals, such as emergency and funeral services personnel.

## Conclusions

Overexposure to death, the circumstances of death and decision-making related to dying patients, have all had a significant emotional impact on healthcare and social services professionals, many of whom express the need for psychological help. The emotional impact and anxiety caused by the number of deaths during the pandemic were not influenced by a practitioner's previous experiences of having worked in environments or residences where there is a high mortality rate, because what happened in the pandemic was unlike anything previously experienced by these professionals. The level of emotional involvement and suffering was lower in professionals dedicated to the collection and burial or cremation of those who had passed, as they were able to maintain distance and limit the degree to which they personalized and identified with the deceased.

## Data availability statement

The data supporting this article will be made available by the authors only on request and on a case-by-case basis, for confidentiality reasons.

## Ethics statement

The studies involving human participants were reviewed and approved by Comité de Ética de la Universidad Pontificia Comillas de Madrid. The patients/participants provided their written informed consent to participate in this study.

## Author contributions

CH-F: conceptualization, data curation, investigation, formal analysis, project administration, resources, visualization, and writing. CM-F: methodology, supervision, and validation. All authors contributed to the article and approved the submitted version.

## Conflict of Interest

The authors declare that the research was conducted in the absence of any commercial or financial relationships that could be construed as a potential conflict of interest.

## Publisher's Note

All claims expressed in this article are solely those of the authors and do not necessarily represent those of their affiliated organizations, or those of the publisher, the editors and the reviewers. Any product that may be evaluated in this article, or claim that may be made by its manufacturer, is not guaranteed or endorsed by the publisher.
